# Metabolic Mechanism of Plant Defense against Rice Blast Induced by Probenazole

**DOI:** 10.3390/metabo11040246

**Published:** 2021-04-16

**Authors:** Zhaochen Wu, Guozhen Wang, Borui Zhang, Tan Dai, Anyu Gu, Xiaolin Li, Xingkai Cheng, Pengfei Liu, Jianjun Hao, Xili Liu

**Affiliations:** 1 College of Plant Protection, China Agricultural University, Beijing 100193, China; zhaochenwu@cau.edu.cn (Z.W.); wangguozhen@csu.edu.cn (G.W.); zhangbr96@cau.edu.cn (B.Z.); daitan@cau.edu.cn (T.D.); cxkxinlang@sina.com (X.C.); seedling@cau.edu.cn (X.L.); 2 Institute of Food Crops, Yunnan Academy of Agricultural Sciences, Kunming 650205, China; ynzycxtd_gu@sina.com (A.G.); lixiaolin@yaas.org.cn (X.L.); 3 School of Food and Agriculture, University of Maine, Orono, ME 04469, USA; jianjun.hao1@maine.edu

**Keywords:** probenazole, metabolomics, systemic acquired resistance, salicylic acid, *Magnaporthe grisea*, gas chromatography, mass spectrum

## Abstract

The probenazole fungicide is used for controlling rice blast (*Magnaporthe grisea*) primarily by inducing disease resistance of the plant. To investigate the mechanism of induced plant defense, rice seedlings were treated with probenazole at 15 days post emergence, and non-treated plants were used for the control. The plants were infected with *M. grisea* 5 days after chemical treatment and incubated in a greenhouse. After 7 days, rice seedlings were sampled. The metabolome of rice seedlings was chemically extracted and analyzed using gas chromatography and mass spectrum (GC-MS). The GC-MS data were processed using analysis of variance (ANOVA), principal component analysis (PCA) and metabolic pathway elucidation. Results showed that probenazole application significantly affected the metabolic profile of rice seedlings, and the effect was proportionally leveraged with the increase of probenazole concentration. Probenazole resulted in a change of 54 metabolites. Salicylic acid, γ-aminobutyrate, shikimate and several other primary metabolites related to plant resistance were significantly up-regulated and some metabolites such as phenylalanine, valine and proline were down-regulated in probenazole-treated seedlings. These results revealed a metabolic pathway of rice seedlings induced by probenazole treatment regarding the resistance to *M. grisea* infection.

## 1. Introduction

Plant diseases are a constraint factor in agricultural production, such as rice blast (*Magnaporthe grisea*) [1], resulting in significant yield and economic losses. In controlling these diseases, fungicides have been used as a major strategy in the production. Most pesticides affect plant diseases in a direct way by inhibiting the pathogen’s growth and biology, but some chemicals may also have effects in an indirect way by inducing plant resistance to pathogen infection [2,3]. Plants have various inherent mechanisms, such as producing defense-related chemicals to protect themselves from biological stresses and potential microbial pathogens. The well-studied systemic acquired resistance (SAR) confers resistance in plants to a broad spectrum of pathogens, concomitant with an increase in chemical inducers such as salicylic acid. Salicylic acid is a constitutive defense compound in plants and a major signal molecule for inducing SAR. SAR-induced resistance in hosts shows effectively inhibitory effects against various pathogens and pests. An apparent and sufficient increase in endogenous salicylic acid inducing the production of pathogenesis-related (PRs) genes are considered as a possible signal function [4,5,6,7]. For example, salicylic acid in rice is 50 times higher than its basal level in responding plant infection and results in high resistance to plant disease [8].

Since probenazole (3-allyloxy-1,2-benzisothiazole-1,1-dioxide) induces SAR in rice, it has been widely used as a plant-defense activator against rice blast for more than 30 years [3]. Despite its extensive use, the development of probenazole resistance in target pathogens has not been observed [9]. According to our observation, probenazole does not provide a strong inhibition on conidial germination against *M. grisea*, nor inhibit mycelial growth. However, probenazole-treated rice or other plants was highly resistant to bacterial leaf blight [10,11]. Thus, probenazole is thought to function either as an SAR activator, like benzo (1,2,3) thiadiazole-7-carbothioic acid S-methyl ester (BTH) and 2,6-dichloroisonicotinic acid (INA), or as a priming effector that enhances defense response activation following pathogen infection [12]. Probenazole has effects in controlling bacterial blight through affecting the stomata of cabbage [13]. Probenazole and its active metabolites 1, 2-benzisothiazol-3 (2H)-one 1,1-dioxide (BIT) act as a chemical inducer of SAR by stimulating a site upstream of the point of accumulation of salicylic acid in the SAR-signaling pathway [14]. BIT and presumably probenazole activate defense responses via the SA/NPR1 signaling pathway in Arabidopsis, tobacco, cucumber and rice [2,5,6,7,8,14]. Probenazole induces *Arabidopsis thaliana* resistance by activating SA-signaling coupled with a consistent increase in callose deposition, which is determined by examining SA-marker genes *PR1* and *PR2* and suppressing JA/ET-signaling determined by examining JA-marker genes *VSP2*, *LOX2* and *PDF1.2* [15]. However, the mechanism of the interaction between rice and probenazole remains unclear.

Metabolomics has been used to determine the relationship between signal transduction, primary metabolites and secondary metabolites in pathogen and host plant interaction [16]. In responding to pathogen infection, defense-related substances in the signaling pathway of plant cells change, such as salicylic acid and ethylene; some key substances also change, such as nitric oxides, ethylene, methyljasmonic acid or methylsalicylic acid to regulate the pathway of SAR, coupled with the change of plant structure and secondary metabolites [5,6,7,8,13]. All these changes contribute to the adjustment on physiology or morphology in hosts corresponding the invasion of pathogens.

The interaction between plant and pathogen can be examined through the change of metabolites. Similarly, the effect of plant defense inducer can be determined by analyzing metabolic pathways. The results will provide reference for understanding the mechanism, interpreting the interaction and searching for potential disease- and defense-related proteins. In our previous study, the non-inhibition of probenazole was tested on *M. grisea* in vitro. Thus, the objectives of this study were to examine the effect of probenazole on rice plants in defending *M. grisea*, profile the metabolome and determine compounds as well as the metabolic pathway related to induced resistance in rice under probenazole treatment.

## 2. Results

### 2.1. Effect of Probenazole on the Metabolome of Rice Seedlings

**Figure 1:**
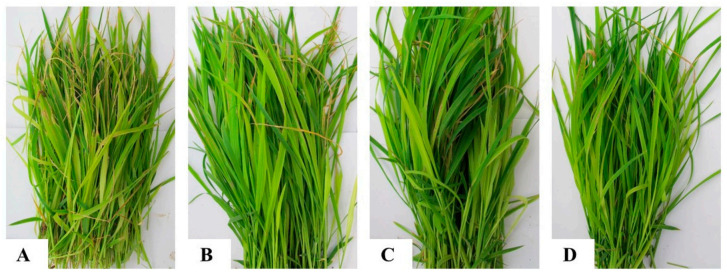
Rice seedlings either untreated (**A**) or treated with probenazole at 75.00 g·m^−2^ (**B**), 112.50 g·m^−2^ (**C**), and 150.00 g·m^−2^ (**D**), followed by inoculation with *Magnaporthe grisea* five days after the treatment.

**Figure 2:**
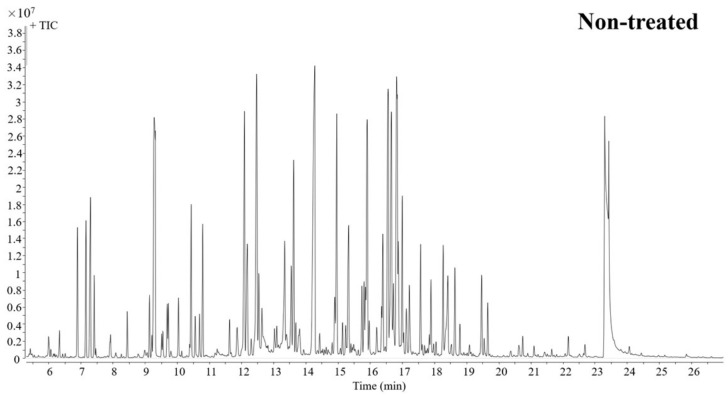

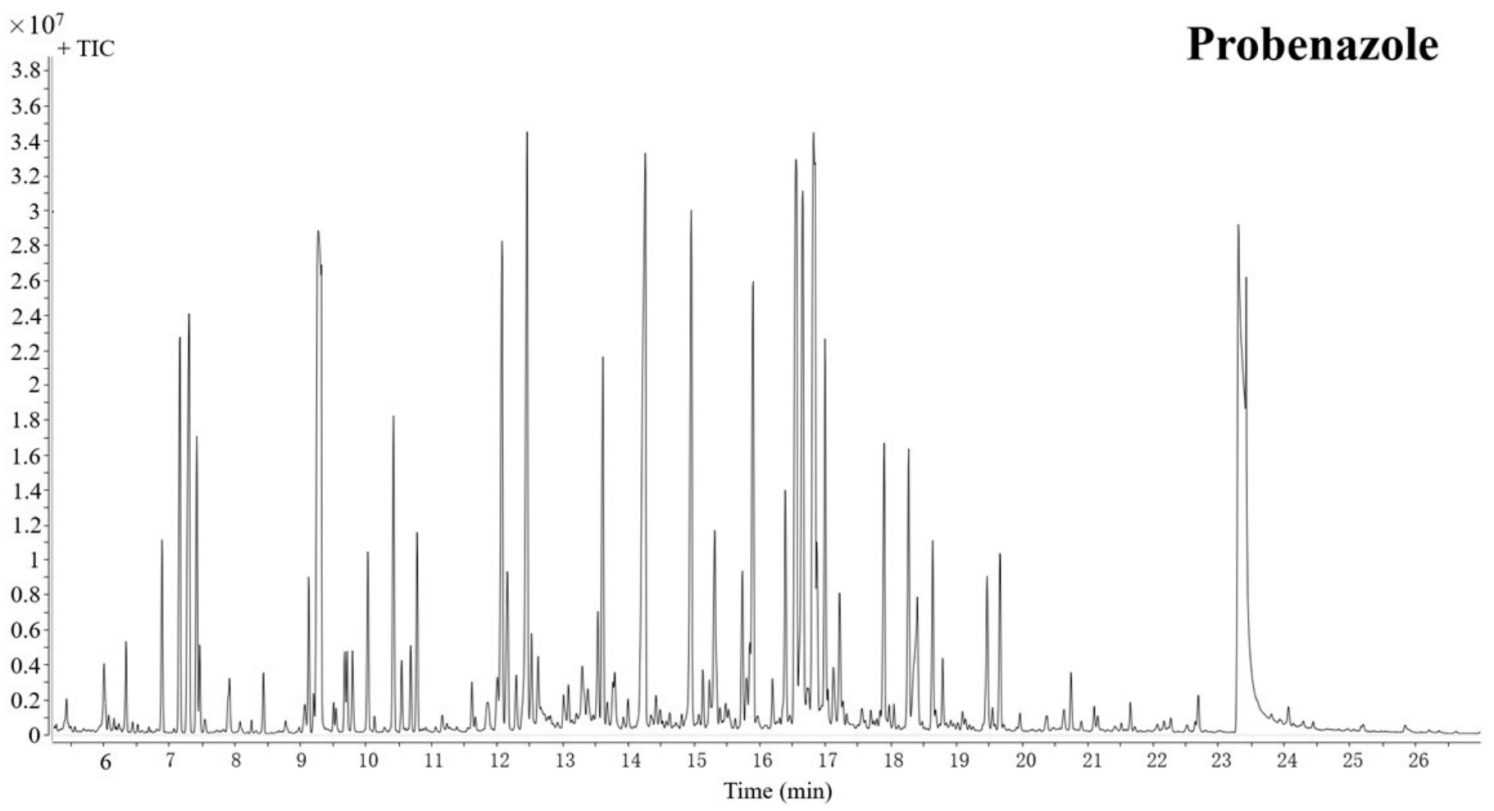
Total ion current diagram of gas chromatography–mass spectrometry on metabolites of rice seedlings either non-treated or treated with probenazole followed by inoculation with *Magnaporthe grisea* five days after the treatment.

**Figure 3:**
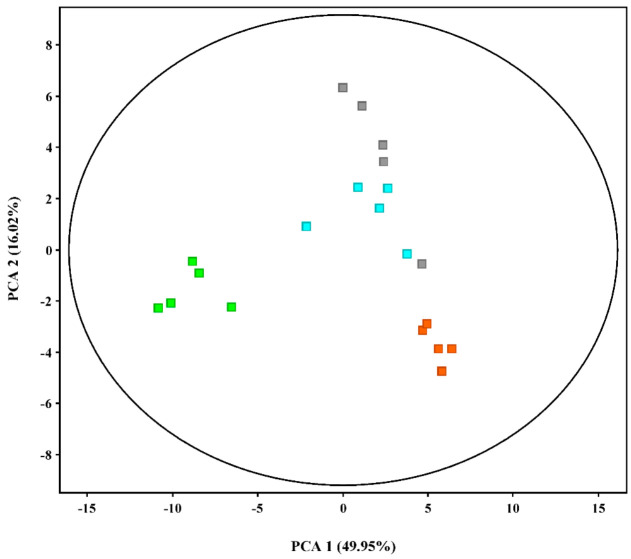
Principle component analysis (PCA) scores of metabolomes in rice seedlings inoculated with *Magnaporthe grisea* without chemical treatment (A group, ■) or treated with probenazole at 75.00 g·m^−2^ (B group, ■), 112.50 g·m^−2^ (C group, ■), and 150.00 g·m^−2^ (D group, ■).

Through *M. grisea*-inoculation coupled with probenazole treatment, the disease index of the control group is 51.29 ± 5.02, and treated groups are 48.4 ± 1.38, 36.59 ± 2.9, and 31.09 ± 6.78, respectively (Figure 1), which has been previously published [17]. The metabolome of *M. grisea*-inoculated rice seedlings with or without probenazole treatments was analyzed on GC-MS (Figure 2). After deconvolution, about 300 peaks were obtained. The metabolome was analyzed using PCA. On the score plot (Figure 3), the probenazole-treated group was distinctively separated from the non-treated group, indicating that probenazole significantly affected the metabolic profile of rice seedlings. This effect was proportionally leveraged with the increase in probenazole concentration. Furthermore, the distance between different biological samples within the same sample was relatively small, while the distance between non-treated and probenazole-treated sampleswas relatively large.

### 2.2. Effect of Probenazole on Differential Metabolites of Rice Seedlings

Metabolites of rice seedlings were detected by GC-MS and compared between probenazole-treated and non-treated samples. A total of 54 metabolites showed obvious change in the three probenazole-treated groups compared with the non-treated group, which were considered closely related to probenazole-induced rice resistance to pathogen infection. Differential metabolites were analyzed against the Fiehn library and NIST library (Supplementary Table S1). All compounds were identified, and the determination was made according to the retention time of the Fiehn spectrum library. All the 54 different metabolites obtained included organic acids, sugars, amino acids, organic alcohols, glycosides, sterols and other categories. In probenazole-treated rice seedlings, 42 metabolites were all up-regulated in rice seedlings treated at three rates. The up-regulated metabolites included salicylic acid, phosphonic acid, aspartate, malate, alanine, fructose, γ-aminobutyric acid, glycine and other important metabolites. Meanwhile, seven metabolites were constantly down-regulated, including terephthalic acid, valine and proline (Supplementary Table S1). The rest of the five metabolites had no constant change directions under different rates of chemical treatment. Of the metabolisms involved in the salicylic acid pathway, shikimic acid was up-regulated but phenylalanine was down-regulated, whereas its downstream metabolite salicylic acid showed up-regulation under the probenazole treatment.

### 2.3. Effect of Probenazole on Metabolic Pathways of Rice Seedlings

**Figure 4:**
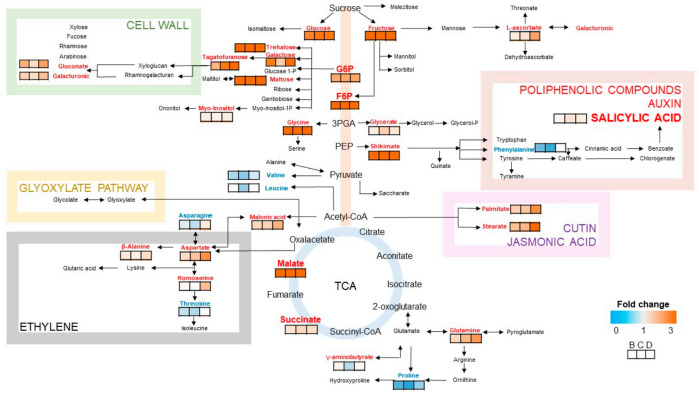
Differential metabolic pathways in rice seedlings related to plant resistance against pathogens. Color panels display fold changes of differential metabolites in rice seedlings treated with probenazole at 75 g·m^−2^ (B group), 112.5 g·m^−2^ (C group), and 150 g·m^−2^ (D group) compared to non-treated group (A group), respectively. Metabolites in bold font mean a significant (*p* < 0.05) differential compared to non-treated group, and metabolites in grey mean non-significant differential.

The metabolic pathways in rice seedling were analyzed using the platform at Metabo Analyst (https://www.metaboanalyst.ca/home.xhtml, accessed on 15 April 2021). Compared with the non-treated plants, for the probenazole-treated plants, a total of 32 out of 54 differential metabolites were displayed in the network of plant metabolic pathways [18] as Figure 4. The schematic indicated a global disturbance in the rice metabolome under the action of probenazole. The results showed that the regulated metabolites were involved in 44 pathways, among which, 12 had an impact greater than 0.1, such as phenylalanine, glycine, serine and threonine metabolisms, arginine biosynthesis, and alanine, aspartate and glutamate metabolisms (Table 1).

## 3. Discussion

The fungicide probenazole was effective in the control of rice blast, but the effect is not via a direct inhibition of pathogen growth. Instead, it affects disease development in an indirect way by inducing plant resistance [14]. We partially confirmed this in our previous work [17]. We have further elucidated the mechanisms of inducing plant resistance in this study, based on the analysis of metabolic profile of rice seedlings. We have found that salicylic acid and serotonin were up-regulated under probenazole treatment. We speculate that salicylic acid induced the expression of pathogenesis-related genes and enhanced the ability of plants in resisting pathogens, and higher content of salicylic acid indicated a higher level of induced resistance in plant.

Salicylic acid participates in the regulation of various physiological and biochemical processes in plants, such as plant flowering, heat production, seed germination, stomata closure, membrane permeability and ion absorption, shown in a previous study [19]. More importantly, it activates the programmed cell death of plants, resulting in necrotic spots at the infection site to prevent further invasion by pathogens [20]. Serotonin participates in various defense reactions of hosts, including programmed cell death, free radical scavenging, and the production of antibacterial metabolites [21]. All these evidences indicated that probenazole induced blast resistance in rice.

Shikimate and phenylalanine are initial precursors in the phenylpropanoid pathway, which is related to plant defense, and strengthens plant cell walls to prevent the colonization of pathogens [22,23]. Since the accumulation of shikimate was up-regulated but phenylalanine was down-regulated, whereas their downstream metabolite salicylic acid showed an up-regulation, it is speculated that probenazole might initiate the transformation of the chemical process. This result was in agreement with other reports that the accumulation of salicylic acid is stimulated by probenazole treatment [9,14]. The overexpression of phenylalanine ammonia-lyase, a key enzyme for salicylic acid synthesis [24], in the probenazole treatment is considered to contribute to wheat resistance to pathogens [25,26]. Besides, whether the other enzymes in the salicylic acid biosynthesis pathway have been strengthened still need to be proven in future research.

Defense responses via jasmonic acid can be activated in response to the infection of necrotrophic pathogens in host plants through wound signaling molecules [27,28]. It participates in abiotic stress responses including drought stress as well [29,30]. Stearate and palmitate increased in probenazole-treated rice seedlings, both of which were precursors of jasmonic acid. Meanwhile, octadecanoid intermediates might participate in a lipid-based signaling system related to jasmonic acid that activates proteinase inhibitor synthesis in response to pathogen attack [31] and this compound was distinctly increased in probenazole-treated rice seedlings.

Pathogen infection affects energy consumption and the production of carbon sources [32]. Sucrose, glucose and fructose are crucial carbon elements in plant photosynthesis. These compounds make syntheses of storage reservoirs used for plant development [33]. We have previously observed a significant increase in sucrose content in pathogen-inoculated leaves of strawberry [34]. Invertase activity increases in response to the infection of powdery mildew in barley, resulting in the accumulation of sucrose and decreased photosynthetic activities [35]. These compounds all increased in probenazole-treated rice seedlings compared to the non-treated group, indicating that probenazole helped plants in resistance against the pathogen.

The glycolysis pathway and tricarboxylic acid (TCA) cycle are involved in mediating respiration homeostasis by generating energy and carbon skeletons that are necessary for biosynthesis, cellular maintenance, and active transport in plants, as well as its relationship with mitochondrial electron transport chain flexibility [36,37]. We observed that glucose, fructose, D-glucose-6-phosphate, fructose-1,6-diphosphate, malate and succinate were all significantly increased in probenazole-treated rice seedlings, suggesting that probenazole could enhance important metabolic pathways in plants.

Cell wall-associated plant defense is an important basal resistance [38]. The components of cell walls are cross-linked by both ionic and covalent bonds into a network that strengthens plant resistance. This development involves the conjugate of xylose, fucose, arabinose, galacturonic and gluconate [39]. The rapidity of the cross-linking of abundant cell-wall-structural proteins makes a rapid defense mechanism to toughen the cell wall as a barrier to pathogen ingress prior to the deployment of transcription-dependent defenses [29]. Therefore, cell walls with highly deposited callose possess resistance to pathogen penetration [40]. SA promotes the increase in lignin to synthesize deposition on the cell wall through the shikimic acid pathway. This lignification enhances the mechanical strength and reduces the degradation of the cell wall by extracellular enzymes from pathogens. The sensitivity and lignification of plants to hyphae or toxins released from pathogens prevent further penetration and infection of pathogens [32]. In our study, we found that compounds involved in the pathway of defense-related cell wall structures including gluconate and galacturonic were all up-regulated in rice seedlings. One possible reason was that most of the above compounds were over synthesized and prepared for cell wall construction in host plants.

Ethylene plays important roles in stress responses [41,42,43,44], growth and development [45] and senescence [46]. Enhanced ethylene production is an early and active response of plants to the perception of pathogen attack and is associated with the induction of defense reactions [47]. Ethylene is suggested to act as a signal involved in SAR [48,49]. The ripening process of climacteric fruit is accompanied by a peak in ethylene production and thus results in a dramatic decrease in fruit hardness [50]. Salicylic acid can inhibit ethylene formation from 1-aminocyclopropane-1-carboxylic acid (ACC) [51]. In this study, ethylene and its precursors were not detected, while the metabolites malonic acid, asparagine, homoserine, aspartate and alanine decreased, compared with threonine, which decreased in the ethylene pathway in rice seedlings. We interpreted that the up-regulation of salicylic acid inhibited ethylene production and therefore sustained plant resistance [51].

Under environmental stresses, γ-aminobutyrate rapidly accumulates and involves itself in the expression of genes for plant signal transduction, transcriptional control, hormone biosynthesis and reactive oxygen species generation and polyamine metabolism, resulting in chemical responses through mitigating stress and enhanced plant resistance [52,53,54,55]. We have shown that the up-regulated glutamine and γ-aminobutyrate were associated with blast resistance in probenazole-treated rice seedlings. Therefore, γ-aminobutyrate is an important metabolite for disease resistance.

## 4. Materials and Methods

### 4.1. Chemical, Plant and Fungal Strain

Probenazole (a.i. 96.8%) was provided by Jiangsu Heyi Chemical Co., Ltd. (Jiangsu, China). Methanol (HPLC grade) was purchased from Sinopharm Chemical Reagent Co., Ltd. (Shanghai, China). Pyridine, methoxyamine hydrochloride, N, O-bis(trimethylsilyl)trifluoroacetamide (BSTFA) (containing 1% trimethylchlorosilane, TMS) were purchased from Sigma-Aldrich (St. Louis, MO, USA). The above reagents are all analytical grades. Ultrapure water was obtained from a Milli-Q system (Millipore, Billerica, MA, USA).

Rice seedlings were grown in 0.15 m^2^ plots in a greenhouse from 25 September to 10 October 2018. At 15 days after emergence, the plants were treated with probenazole in granular formulation (a.i. 16%) at different rates, including A: 0, B: 75.00 g·m^−2^, C: 112.50 g·m^−2^, and D: 150.00 g·m^−2^ (Table 2). The choice of high and low concentrations is within the recommended dose (http://www.chinapesticide.org.cn/, accessed on 19 March 2021). Five days after chemical treatment, the seedlings were inoculated by spraying 2 × 10^5^ mL^−1^ spore suspension of *M. grisea*. Water mist was sprayed for 2 min per hour to keep a high moisture. Disease was evaluated and 100 rice seedlings per plot were randomly sampled at 7 days post incubation. The samples were kept in a Ziplock bag and stored at −80 °C for later analysis.

### 4.2. Pretreatment

The sample of rice seedlings was processed following the procedure of metabolome analysis according to Dai et al. [56]. Briefly, the sample was pre-chilled with liquid nitrogen and ground in a ball mill (MM400, Verder Shanghai Instruments and Equipment Co., Ltd., Shanghai, China) at 30 Hz for 1 min. For each treatment, five samples were collected as replicates, 100 ± 1 mg sample was weighed and dissolved in 1.8 mL of extraction solvent (methanol/water, *v*/*v* = 8/2) with 10 μg∙mL^−1^ ribitol as internal standard with 5 replications (in 5 tubes). The sample was treated with 100 Hz ultrasonic for 20 min, followed by centrifugation at 13,800× *g* for 15 min. Immediately, 0.4 mL of supernatant was pipetted and desiccated at 45 °C in a vacuum concentrator and stored at −20 °C until use. Derivatization was performed in two steps. (1) An aliquot of 100-μL methoxyamine hydrochloride solution at 20 mg∙mL^−1^ was added to the sample and incubated at 30 °C for 2 h. (2) The sample was added with 100 μL containing BSTFA (1% TMS) and incubated on a dry bath block at 37 °C for 6 h. After centrifugation, 120 μL of liquid supernatant was transferred into a sample vial sealed with a rubber cap, and metabolome detection was performed within 48 h.

### 4.3. Gas Chromatography and Mass Spectrometry (GC-MS) Analysis

Rice metabolites were separated and detected using an HP-5MS capillary column (30 m × 0.25 mm × 0.25 μm) coupled with the 7890A-5975C GC-MS system (Agilent, CA, USA). Helium was used as carrier gas with a 1.1 mL∙min^−1^ flow rate. Each injection volume was 1 μL. A GC oven was heated to 60 °C for 1 min, raised to 325 °C at 10 °C/min for 2 min. The auxiliary heater was 290 °C. The ion source (EI) temperature was set to 250 °C. Electron impact ionization (70 eV) was set in a full scan mode (*m*/*z* 50 to 600) to 0.2 s/scan.

### 4.4. Data Analysis

MassHunter Qualitative Analysis B.07.00 (Agilent Technologies, Santa Clara, CA, USA) was used for data processing and deconvolution with the parameter of 30000 absolute peak height, and NIST14 and Fiehn mass spectrometry databases were used as references for qualitative analysis. Agilent MassHunter Mass Profiler Professional 13.1.1 was used for principal component analysis (PCA), cluster analysis and variance analysis. Metabo Analyst online (https://www.metaboanalyst.ca/home.xhtml, accessed on 15 April 2021) analysis software was used to conduct metabolic pathway analysis. Analysis of variance (ANOVA) was performed, and metabolites were compared between probenazole-treated and non-treated groups. Significant difference was determined with the fold change >1 and *p* < 0.05.

## 5. Conclusions

Probenazole application induced rice resistance to rice blast, which was confirmed by significant metabolic changes, including up-related salicylic acid, γ-aminobutyrate and shikimate while there was down-regulated phenylalanine, valine and proline. These might be related to the strengthened enzymes, such as phenylalanine ammonia-lyase in the salicylic acid pathway. As such, plant protection in the phenylpropanoid pathway effectively enhanced the defense ability of rice. This work will enrich the understanding on the mode of action of probenazole in a salicylic acid-mediated plant immune system.

## Figures and Tables

**Table 1:** Differential metabolite of rice seedlings treated with probenazole, showing pathways containing more than two compounds.

Pathway	Match Status ^a^	P ^b^	Holm P ^c^	Impact ^d^
Ascorbate and aldarate metabolism	1/4	0.016	0.196	0.500
Phenylalanine, tyrosine and tryptophan biosynthesis	1/4	0.097	0.582	0.500
Alanine, aspartate and glutamate metabolism	3/14	0.000	0.002	0.424
Phenylalanine metabolism	1/10	0.226	0.945	0.357
Glycine, serine and threonine metabolism	1/11	0.048	0.443	0.270
Arginine biosynthesis	3/14	0.004	0.093	0.228
Glyoxylate and dicarboxylate metabolism	1/8	0.007	0.125	0.185
Inositol phosphate metabolism	1/15	0.173	0.807	0.129
Pentose and glucuronate interconversions	1/18	0.370	1.000	0.125
Starch and sucrose metabolism	1/9	0.073	0.559	0.123
Tryptophan metabolism	1/41	0.653	1.000	0.105
Arginine and proline metabolism	1/19	0.248	0.945	0.102
Glycerolipid metabolism	1/16	0.336	1.000	0.093
Galactose metabolism	1/9	0.028	0.298	0.092
Glutathione metabolism	1/28	0.513	1.000	0.089
Citrate cycle (TCA cycle)	1/10	0.088	0.570	0.077
Glycolysis / Gluconeogenesis	1/13	0.137	0.702	0.072
Fructose and mannose metabolism	1/20	0.401	1.000	0.051
Pentose phosphate pathway	3/22	0.016	0.196	0.047
Phosphatidylinositol signaling system	1/28	0.513	1.000	0.037
Butanoate metabolism	2/15	0.053	0.443	0.032
Pyruvate metabolism	1/22	0.431	1.000	0.031
Fatty acid biosynthesis	1/47	0.704	1.000	0.015
Glycerophospholipid metabolism	1/36	0.605	1.000	0.013
Primary bile acid biosynthesis	1/46	0.696	1.000	0.008
Tyrosine metabolism	1/42	0.662	1.000	0.007
Amino sugar and nucleotide sugar metabolism	2/37	0.238	0.945	0.000
Aminoacyl-tRNA biosynthesis	5/24	0.000	0.000	0.000
beta-Alanine metabolism	1/21	0.416	1.000	0.000
Biosynthesis of unsaturated fatty acids	1/18	0.229	0.945	0.000
D-Glutamine and D-glutamate metabolism	1/6	0.142	0.702	0.000
Fatty acid degradation	1/39	0.634	1.000	0.000
Fatty acid elongation	1/39	0.634	1.000	0.000
Histidine metabolism	1/16	0.336	1.000	0.000
Lysine degradation	1/25	0.474	1.000	0.000
Nicotinate and nicotinamide metabolism	1/15	0.319	1.000	0.000
Nitrogen metabolism	1/6	0.142	0.702	0.000
Pantothenate and CoA biosynthesis	2/19	0.081	0.564	0.000
Porphyrin and chlorophyll metabolism	1/30	0.538	1.000	0.000
Propanoate metabolism	1/23	0.446	1.000	0.000
Purine metabolism	1/65	0.816	1.000	0.000
Pyrimidine metabolism	1/39	0.634	1.000	0.000
Selenocompound metabolism	1/20	0.401	1.000	0.000
Valine, leucine and isoleucine biosynthesis	3/8	0.001	0.021	0.000
Valine, leucine and isoleucine degradation	1/20	0.267	0.973	0.000

^a^ Match status is the number of matching metabolites over the total number of metabolites. ^b^ P: probability of enrichment analysis; ^c^ Holm P is probability adjusted by Holm Bonferroni method; ^d^ impact is the path topology value path influence.

**Table 2:** Grouping of probenazole-treated rice seedlings inoculated with *Magnaporthe grisea*.

Pesticide	Dispose Group	Gram/m^2^
None	A	0
16% probenazole granules	B	75.00
16% probenazole granules	C	112.50
16% probenazole granules	D	150.00

## Data Availability

The data presented in this study are available in insert article and Supplementary Materials here.

## Supplementary Materials

The following are available online at https://www.mdpi.com/article/10.3390/metabo11040246/s1, Table S1: Changes in rice plants metabolites after exposure to probenazole.
