# Supplementary material for: Metabolic Mechanism of Plant Defense against Rice Blast Induced by Probenazole
Source: Metabolites. 2021 Apr 16;11(4):246. doi: 10.3390/metabo11040246 (PMC8073365; doi:10.3390/metabo11040246)
Supplement: Supplementary file 1 [file metabolites-11-00246-s001.zip › metabolites-1146011-supplementary.pdf]

Supplementary Table **Changes in rice plants metabolites after exposure to probenazole**

**Metabolites** [a]	**RT (min)** [b]	**M/Z** [c]	**Score values**	**Fold Change** [d]		
				**B vs A**	**C vs A**	**D vs A**
Valine	8.44	144	94.80	0.73	0.63	0.86
Malonic acid	8.78	73	63.42	1.53	1.59	2.06
Ethanolamine	9.13	174	94.40	1.14	1.27	1.39
Leucine	9.20	158	82.70	0.98	0.66	1.01
Phosphonic acid	9.28	300	88.80	1.24	1.69	1.45
Proline	9.55	142	93.40	0.47	0.39	0.66
Glycerate	10.03	73	94.40	1.25	1.66	1.36
Citrulline	10.55	141	53.70	0.60	0.58	0.76
Threonine	10.79	73	95.50	0.80	0.74	1.03
4-Ketoglucose	10.88	73	58.50	0.80	1.16	1.56
Aspartate	11.17	73	82.90	1.48	1.78	2.48
Malate	12.01	73	89.50	23.93	13.62	40.27
Salicylic acid	12.30	267	85.30	1.13	1.35	1.25
Pipecolic acid	12.48	258	77.60	0.85	0.97	1.23
γ-aminobutyrate	12.51	84	89.80	1.15	0.76	1.09
Homoserine	13.05	218	56.70	1.10	1.06	1.88
Phenylalanine	13.68	218	86.50	0.53	0.43	0.98
L-Alanine	13.77	116	68.93	1.30	1.25	1.45
Cyclooctasiloxane	14.00	355	87.90	4.42	2.66	7.93
Asparagine	14.19	275	89.00	0.89	0.79	1.28
Glutamine	15.31	357	88.40	1.53	1.96	2.37
Tagatofuranose	15.35	217	64.50	2.02	2.80	3.87
Terephthalic acid	15.44	295	69.50	0.93	0.27	0.14
2-Keto-1-gluconic acid	15.56	133	64.10	1.06	2.77	2.61
Shikimic acid	15.73	429	87.20	>100	>100	>100
Fructose	16.31	218	70.00	>100	>100	>100
Glucuronic acid	16.49	288	71.90	1.65	1.33	2.19
α-D-Galactopyranoside	16.72	204	85.00	0.42	0.30	0.31
Ascorbate	17.23	73	59.50	1.28	1.35	2.25
Glycine	17.28	73	83.50	6.43	3.57	11.13
Gluconate	17.75	73	81.20	2.10	1.45	2.28
Palmitate	17.90	313	93.90	1.63	1.77	2.50
Galacturonic	17.98	73	82.00	1.63	1.45	1.89
Succinate	18.27	73	61.80	1.38	1.51	1.47
Myo-Inositol	18.64	305	94.20	1.25	1.17	1.22
Octadecane	18.75	71	63.70	>100	>100	>100
α-D-Glucopyranose	18.80	73	57.00	1.54	1.36	1.65
Phytol	19.09	143	70.60	1.35	1.11	1.93

Maltose	19.45	68	84.60	3.16	3.01	5.59
Stearate	19.67	341	86.60	1.86	1.97	3.02
D-Glucopyranosiduronic acid	19.94	253	65.80	>100	>100	>100
Cyclononasiloxane	19.97	73	79.10	>100	>100	>100
Lactulose	20.47	204	70.20	1.66	1.09	2.77
D-glucose-6-phosphate	20.75	387	91.30	2.15	2.18	2.20
D-Glucose	20.91	73	84.70	2.64	2.83	2.80
Serotonin	21.65	174	81.40	1.58	1.97	1.31
Cellobiose	22.07	204	74.10	2.36	1.75	2.75
3,4-Dihydroxymandelic acid	22.27	73	79.60	>100	>100	>100
Fructose-1,6-diphosphate	22.64	73	87.90	5.44	3.48	4.19
Turanose	22.69	73	79.10	3.03	2.43	2.09
Sucrose	23.59	103	90.10	1.36	2.56	2.61
D-Trehalose	23.81	361	82.80	9.69	7.89	6.85
Aucubin	25.21	73	57.90	>100	>100	>100
Galactose	25.84	204	88.10	2.77	1.67	3.42

[a]A metabolite was selected. If the content changed significantly ($P < 0.05$) in probenazole-treated rice for at least three out of five sampling times compared with non-treated rice. [b]RT = retention time. [c]m/z = mass-to-charge ratio. [d]The metabolites contents changed in probenazole-treated rice seedlings inoculated *Magnaporthe grisea* compared with non- treated ($P < 0.05$). Numbers greater and less than 1 represent an up-regulation and down-regulation of metabolites, respectively.
